# Challenging the assumption that medical conditions are the prime drivers of older people’s needs: a qualitative baseline study about the experienced needs of older people who age in place with COPD

**DOI:** 10.1186/s12877-026-07800-4

**Published:** 2026-06-26

**Authors:** J. M. Bergschöld, S. Bechmann Granås, S. Bergh, L. Dyrendal Høgset

**Affiliations:** 1 Department for Health Research, SINTEF Digital, Oslo, Trondheim, Norway; 2 The Research Centre for Age-related Functional Decline and Disease, Innlandet Hospital Trust, Brumunddal, Norway; 3 Norwegian National Centre for Aging and Health, Vestfold Hospital Trust, Tønsberg, Norway

**Keywords:** User involvement, Gerontechnology, COPD, Co-design, Needs identification, Technology development, Age in place, Older people

## Abstract

**Background:**

In the hope of decreasing the likelihood that older people will reject technologies that are meant to enable them to age in place, the involvement of older people in the development process has become common. Meanwhile, the literature on methods for involving older people is still emerging, and so far, there is no common knowledge base that studies which involve older people in technology design refers to. Yet, it is possible to identify patterned practices, such as the practice of recruiting older people based on their medical condition. This practice assumes that medical conditions are prime drivers for older people’s needs, and that recruiting older people with the same medical condition will therefore lead to the discovery of homogenous needs for technology, thus decreasing the risk of rejection. The aim of this study is to describe the experienced needs of older people who have been diagnosed with Chronic Obstructive Pulmonary Disease (COPD) and age in place in Norway at baseline in an ongoing project.

**Methods:**

In this qualitative interview study we conducted 10 baseline interviews with older adults (65+) with COPD who are ageing in place. While these older people were recruited into the study based on their diagnosis, the interviews encouraged the informants to also describe other challenges and needs that they deal with in the context of their everyday lives.

**Results:**

The results illustrate that Norwegian older people with COPD do share some common challenges related to their medical condition. Most of the problems that they are dealing with, and consider important to solve, have more to do with general problems related to the everyday experience of ageing in a rural community in Norway than with their medical condition.

**Conclusions:**

The article contributes to the methodological literature on involving older people in the development of technology for ageing in place by challenging the assumption that medical conditions are prime drivers of older people’s needs. It illustrates how this assumption may point technology developers in the wrong direction unless it is supplemented by a strong focus on the everyday experience of ageing.

## Background

The EU population is ageing, and projections indicate that the share of people over 65 years could reach 30% of the EU population by 2030, as opposed to 10% in 1960 [1]. This demographic ageing is even more acute in rural areas, where there are now fewer than two people of working age for every elderly person [2]. New technology that enables older people to age in place offer opportunities to alleviate the challenges posed by demographic ageing, especially in terms of health care [3]. Aging in place is a term often defined as continuing to live in the community, with some level of independence, rather than in residential care [4]. However, this requires that the new solutions are seen as attractive, useful, and relevant for older people, who are otherwise likely to reject them [5].

Norway stretches 1,800 km from south to north and has a population of 5,3 million people with the majority living in the south-eastern part [6]. The climate is cold, meaning that even in the south temperatures frequently drop below − 20 °C, and that heavy snowfalls and ice-covered roads is the norm between October and May in large parts of the country. The Norwegian living lab in SMILE is in Gudbrandsdalen. This is a large rural valley in the south-eastern part of Norway that consists of 12 municipalities with 1,950–6,000 inhabitants each, and a city located in the southern part of the valley, with approximately 28,500 inhabitants. The landscape is mountainous and sparsely populated [7]. Houses are typically very old, meaning that they are small and not built according to the principles of universal design which can make them difficult to grow old in [8]. Moreover, they are often located on steep hills and sit on large acreages meaning that distances between people, workplaces, public services, care service, and care institutions are long [9]. For instance, the hospital for the municipalities in this area is in the city, meaning that for many people the distance to the hospital is up to 170 km. In Norway, the number of people over the age of 80 will almost double until 2040 in rural areas [10], meaning that rural areas such as Gudbrandsdalen are facing considerable challenges in the context of demographic ageing.

The study was conducted as part of SMILE, an EU-project that was funded by Horizon 2020. The objective of SMILE was to create smart living environments for older people by developing modern technologies that would enable them to age in place with dignity and on their own terms. To this end, the project has constructed living labs [11] in Norway, Denmark, the Netherlands, and Canada for the purposes of working with older people with dementia, COPD, or post-surgery recovery to co-create digital and related service solutions that address their needs and preferences for independent living.

In this context, the involvement of older people in the development of new technology for ageing in place has become a *sine qua non* [12]. Meanwhile, the literature on methods for involving older people is still emerging, and so far, there is no common knowledge base that studies which involve older people in technology design refers to [13]. Yet, it is possible to identify patterned practices, such as the practice of recruiting older people based on their medical condition. This practice assumes that medical conditions are prime drivers for older people’s needs, and that recruiting older people with the same medical condition will lead to the discovery of homogenous needs for technology, thus decreasing the risk of rejection [14–23].

This article contributes towards the methodological literature on involving older people in the development of technology by illustrating the importance of open exploratory approaches that do not privilege medical challenges over the “mundane” everyday challenges of ageing in place, even in innovation projects that focus on e-health solutions and recruit informants based on their medical diagnoses. Therefore, the aim of this study is to describe the experienced needs of older people who have been diagnosed with Chronic Obstructive Pulmonary Disease (COPD) and age in place in Norway at baseline in an ongoing project.

## Methods

We conducted 10 baseline interviews with older adults (65+) with COPD who are ageing in place in the Gudbrandsdalen area. In similarity with other projects involving older people in the development of technology, their medical diagnosis was used as a criterion for recruitment based on the assumption that this would allow for the identification of a relatively homogenous set of needs that the older people as well as their formal and informal caregivers find important, to increase the likelihood of successful scalability.

Drawing on practical situations that older people face in their daily lives, the main aim of SMILE was to create SMart Inclusive Living Environments (SLE) with novel eHealth solutions that enable ageing in place. In line with this aim, the baseline interviews did not just address the challenges of ageing in place with COPD, but they also encouraged the informants to talk about challenges related to their everyday experience of ageing in place. We applied a qualitative interview study design, where individual semi-structured interviews with the participants were loosely structured and thematically organized around the following questions: What is your everyday situation like? What is important for you in your everyday context? What technologies or other devices do you use to deal with challenges related to COPD? How does your contact with healthcare services and people who care for you look like today? What are your wishes and concerns in relation to ageing in place in the coming years? The interviews were conducted during the autumn of 2021 and the early spring of 2022. In total, we conducted 10 baseline interviews with 10 older adults that have been diagnosed with COPD and are ageing in place in Gudbrandsdalen. Each interview lasted for approximately one hour. After each interview the researchers engaged in debriefing discussions. The identification of our main analytical finding, that problems related to COPD were not the only or even the most important challenges that the informants described in in the baseline interviews first emerged iteratively during these debriefing discussions. All interviews were audiotaped and transcribed *ad verbatim*. Transcriptions were anonymized by swapping the informants real names for random Norwegian names. Moreover, the names of the small rural hamlets where the informants live and the municipalities where they are located are omitted and only the county is named. Quotations presented in this article have been translated from Norwegian to English by the authors. All translations privilege clarity of meaning over the verbatim.

In analysing the transcriptions, we drew on techniques from social constructionist grounded theory [24–26]. First, the transcriptions were coded using in vivo coding meaning that each segment where an informant talked about a challenge the entire segment was turned into a code [27]. Thereafter the in vivo codes were sorted into groups. This sorting process involved adding all vivo codes to the network view in Atlas.ti and considering them in relation to each other for the purposes of identifying emergent categories and themes that could be used to describe and group the in vivo codes.

The analysis identified four main themes in the informants’ descriptions of everyday challenges related to ageing in place. Each main theme encompasses several categories. Each category consists of at least one in vivo code. The in vivo codes are presented as quotes in the descriptive presentation of the analysis in the following section.

## Results

**Fig. 1:**
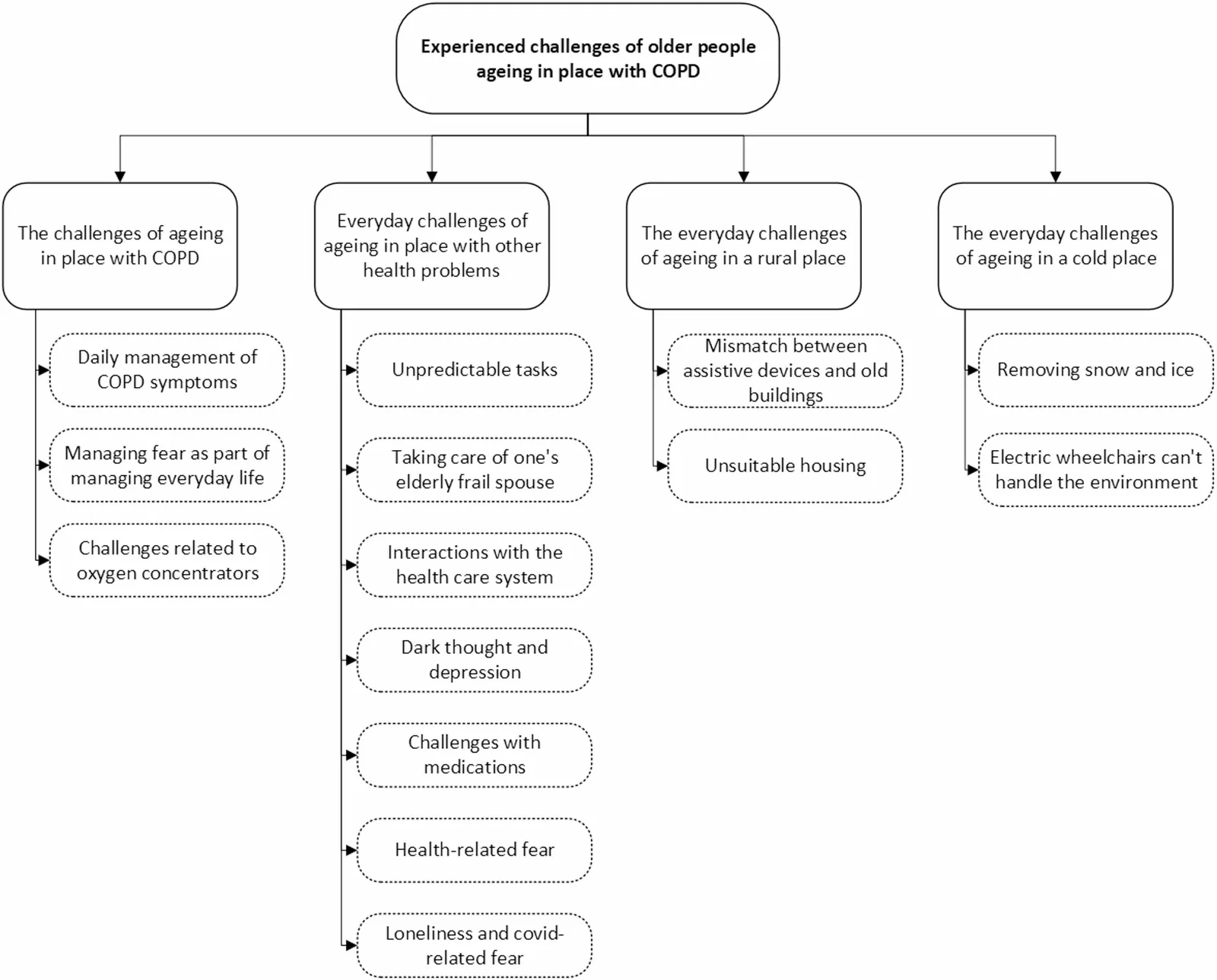
Overview of over-arching themes and subthemes

The analysis identified four overarching themes that illustrates the participants’ experienced challenges of ageing in place. These themes are (1) challenges related to living with and managing COPD, (2) challenges related to other health problems, (3) challenges related to living in a rural environment, and (4) challenges related to living in a cold Norwegian climate. Each theme consists of several subthemes. Together, these illustrate that while COPD shapes certain aspects of daily life, many challenges described by the participants stretches into broader health, social and environmental contexts (Fig. 1).

### The challenges of ageing in place with COPD

In talking about the challenges of ageing in place explicitly related to COPD, the informants’ descriptions highlighted a variety of issues. Specifically, daily management of COPD Symptoms, managing fear as part of managing everyday life, unexpected consequences of COPD, night-time challenges, and challenges related to assistive devices.

### Daily management of COPD symptoms

The informants described that while they depend on to use medicines and assistive devices to manage their COPD symptoms, they felt that this was something they mostly managed without issues and did not perceive as challenging.

For instance, Ellen described that while she used to have trouble sleeping, she now has an oxygen concentrator that she uses during the night. This had improved her symptoms and overall wellbeing.

*Quote 1: I have a machine*,* an oxygen concentrator*,* that I use during the night*,* and it has worked wonders for me. I haven’t felt so bad after I got it. I sleep well during the night and I’m not so tired during the day*,* so I don’t really feel like I am ill.*

#### Remembering to take and to always carry one’s medicine

Descriptions about the informants’ everyday management of COPD symptoms also concerned solutions in the form of daily routines that they have created for themselves, which enable them to remember taking their medicines and to bring their medicines if they leave the home.

### Daily routines

For instance, Marie described how she has created daily routines for herself to make sure she never forgets her regular medicines, she always takes them when she goes to the bathroom in the morning and if she’s going somewhere she makes sure to put them in her pocket or in her bag. Similarly, Kirsti described that she has created a routine where she takes her medicines during breakfast and after dinner when she is going to bed.

*Quote 2: I take my pills first thing in the morning*,* or… No*,* I don’t. First*,* I go to the kitchen where I make myself a sandwich and then I bring it back to bed where I eat it and drink a glass of milk*,* THEN I take them. And during the evening*,* I eat my supper and then I go to bed. Well first I take care of things in the bathroom and then I take them. Right before I get into bed. So that’s a set routine. I always do it in the exact same way*,* so I don’t need any reminders like “now you need to take your medicine”.*

### Managing fear as part of managing everyday life with COPD

Many informants expressed that they frequently experience COPD related fear and that they have devised methods of managing this fear. Such methods range from closely monitoring their own health values, to actively avoiding keeping informed about their health values.

#### Fear caused by not knowing one’s measurements

The informants described that they have constructed methods of monitoring and avoiding measuring health values related to their COPD as a way of making their fear manageable. Together, these descriptions are illustrative of how some older people with COPD experience a need for technology that allows them to register their own health values, whereas others experience a strong need to avoid knowing their own health values.

For instance, Frode described how not knowing certain health measurements caused anxiety, especially for developing pneumonia. This led him to worry a lot:

*Quote 3: It’s very tiring*,* because it’s constant*,* you’re thinking about it all the time. Am I catching pneumonia now? Is the INR value too high so that I’ll bleed?*

Similarly, Ellen described that she has bought a pulse oximeter which she always keeps close and uses to measure her values whenever she feels out of breath, or that her heart is racing. Especially when she has a cold. While she doesn’t make a note of her values or keep a record of any kind, just knowing that she can measure her values to see whether they are within acceptable levels, or if she needs to call for help, is calming to her.

*Quote 4: This [pulse oximeter] measures the level of oxygen in the blood and the pulse. […] I don’t note down the results [but] If I feel that my pulse goes up*,* I can just put it on my finger and then I know that the level of oxygen isn’t so bad*,* and that I’m ok. […] if not*,* I can contact someone for help.*

#### Fear caused by knowing one’s measurements

Contrarily, other informants described that they actively avoided measuring their values as a way of managing their fear. These informants described how constantly being aware of their measurements was both depressing and created a lot of fear. By actively avoiding knowing their measurements, they were able to manage their fears.

For instance, Sandra described how she has bought a pulse oximeter that she uses if she feels very out of breath, as a way of measuring whether she needs help. For her, this is typically in conjunction with going to the bathroom which is a physically demanding task for her. But while she does measure her values, she emphasizes that she does not document the results and that she actively avoids using the pulse oximeter outside of these situations as she feels that this could create fear.

Quote 5: *I don’t [use it a lot]*,* I just use it to check [my values if I feel that I need to]*,* if you use it too often*,* I think it might make you frightened.*

Similarly, Marius pointed out that people who have had the diagnose for a short time has different needs than people like him who have lived with the diagnosis for a long time.

Quote 6: *In the beginning you want to keep a close eye on your values because it gives you a sense of control*,* but after a while you understand that there is no controlling it and that you become depressed if you follow your own values too closely. They will only go one way”.*

#### Struggling to fall asleep due to exacerbated symptoms that cause anxiety and fear of dying

Similarly, while most describe that the day-time medical management of their COPD symptoms is a challenge that they feel that they can manage with their available resources, many struggle to fall asleep.

For instance, Mari described that she experiences several challenges tied to sleep. Not only is it physically challenging for her to get ready for bed because it involves many movements that affect her ability to breathe. The fact that she typically loses her breath during this process leads to a situation where she thinks a lot about her inability to breathe properly right when she is supposed to relax and be able to go to sleep. Instead, she spends a lot of time lying there, thinking about how heavy her breathing is which often results in strong feelings of anxiety and fear of dying.

*Quote 7: Well*,* that’s the psychological bit of this*,* the fear of dying and all these heavy…heavy thoughts. You dread going to bed. Both on account of losing your breath because it’s hard work to go to the bathroom and get ready for bed*,* and then the thoughts come. Because then you lie there and feel yourself struggle to breathe and you wonder if this is the last night and then… it’s things like that. So*,* there are a lot… a lot of thoughts that shouldn’t be there*,* but they come anyway.*

### Challenges related to oxygen concentrators

In talking about their experiences of being entirely dependent on, or frequently using, oxygen concentrators to manage everyday life. People with COPD sometimes need long-term oxygen therapy/treatment because they are unable to breathe deeply enough. Oxygen concentrators are devices that can provide people who need extra oxygen with a steady supply by selectively removing nitrogen from ambient air. As such, they are constructed specifically for the purpose of addressing the challenges that people with COPD have. Yet, several informants described that, while they are certainly dependent on oxygen concentrators, these devices can also pose challenges of their own. The informants’ descriptions of the challenges that oxygen concentrators pose in the context of their experiences of ageing in place featured descriptions of both stationary and portable devices.

Specifically, the challenges mentioned were *that: portable oxygen concentrators are heavy and therefore difficult to use*,* that portable oxygen concentrators shut down in cold weather*,* and that stationary oxygen concentrators create a risk of tripping and falling.*

### Portable oxygen concentrators are too heavy

Participants described that even portable oxygen concentrators are cumbersome and heavy. For instance, Tine described that her ability of leaving the home depends on her using a portable oxygen concentrator but that she feels that it is too heavy for her to carry. There is a strap on the device that is meant to enable the user to carry it over their shoulder, or across their back like a bag. But because it is so heavy, she puts it in her walker.

Quote 8: *This is like a handbag that I can use and carry like a backpack*,* but I think it’s too heavy so I usually but it in my walker.*

### Portable oxygen concentrators can shut down in cold weather

Tryggve also described how his portable oxygen concentrator has contributed positively to his life and emphasized that not only does it facilitate the management of everyday challenges of ageing in place with COPD. It is the single most important assistive device he has because it enables him to continue participating in society. With a lung capacity of approximately 24% he is not allowed to drive his car without a steady supply of oxygen, due to the risk that he could otherwise become unconscious. Yet, after a frightening episode when the portable oxygen concentrator suddenly shut down due to cold weather conditions, he no longer dares to rely on it during the winter months.

*Quote 9: It was a terribly cold winter; really cold you know. Maybe minus 26–27 degrees Celsius*,* and as I was walking to my car it was as if I suddenly lost my breath*,* and I realized that [the portable oxygen concentrator] had stopped working. As luck has it*,* a nurse came by and was able to get me inside and then [the portable oxygen concentrator] started up again but I thought I was going to die and after that incident I don’t go out when it’s cold.*

### Long tubes increase the risk of falling

Stationary oxygen concentrators are frequent occurrences in the homes of people with COPD. These are larger, heavier, and not meant to move around with the user. Instead, it comes with a long flexible tube so the user can move around freely. Stationary oxygen concentrators often improve the quality of sleep for people with COPD, yet the informants also described that it could pose risks.

For instance, Harald described how the long tube frequently knocks things over and gets tangled which is very frustrating.

*Quote 10: this one [a stationary oxygen concentrator] runs all the time*,* and it has a long tube that I always must kick out of my way. I’m so annoyed with it*,* it’s almost as if its constantly coiling around itself and then I have to stop what I’m doing and untangle it and then it inevitably gets caught on stuff and I … [he sighs] it’s just difficult to have to deal with it all the time*.

Similarly, Nina emphasized that while a stationary oxygen concentrator is necessary, she is scared by how the long tube is simultaneously constitutive of an ever-present falling hazard,

*Quote 11: a long tube like that is quite scary for someone like me who has difficulties walking in the first place.*

As these descriptions illustrate, oxygen concentrators certainly do help older people with COPD to age in place, but they also pose challenges of their own such as an inability to function in winter temperatures common to the northern hemisphere and a risk of falling.

### The everyday challenges of ageing in place with other health problems

In addition to COPD, all the informants have other health related problems that create challenges that they must manage as part of the everyday reality of ageing in place.

### Unpredictable tasks are more difficult to get help for

In talking about the everyday challenges of other health related issues, one of the informants highlighted how the increasingly stiff joints in her hands make it difficult for her to manage everyday tasks that involve small objects, such as for instance changing the batteries of her hearing aid. While she described that she did have people she was comfortable relying on for help, she also felt that there was a difference between asking for help with tasks such as grocery shopping, getting a ride to an appointment or having her lawn cut, whereas needing help with the management of menial tasks involving small objects such as changing the batteries to her hearing aid, or changing a lightbulb, are more difficult to get help with. Not because she lacks people who are willing to help her, but because these tasks are unpredictable. Asking for and getting help with predictable tasks is easier because then the person helping her can plan for it and set aside a time slot that suits them. But asking for help with short notice makes her feel like she’s a bothersome person who is constantly nagging and asking for things.

*Quote 12: my son and his wife help me by mowing the lawn*,* and the home care services help me to clean the house*,* but it’s really the small unpredictable things that are difficult when you need help. Like changing a blown fuse or a lightbulb. So*,* feel like I’m constantly nagging them.*

### Being elderly, frail, and burdened by the responsibility of caring for one’s elderly frail spouse

The informants also described that while they themselves are older people who depend on care services to age in place, they are simultaneously informal caregivers to their spouses.

For instance, Sigrid described that her husband’s health problems pose great challenges for her and where she is in a situation where she needs support to be able to care for him. While she receives some support from the homecare services, he also has a diagnosis where it is likely that his need for care will increase significantly in the next few years. This creates challenges for her in the form of worry for the future, as well as an extra burden where she feels obligated to care for him.

*Quote* 13 *It’s a huge responsibility*,* […] I don’t want to complain because it’s not so much work*,* but it is a big responsibility*,* [that sometimes weighs on me]*.

### Challenges in interactions with the health care system

Challenges in interactions with the health care system was another important theme in the informants’ descriptions of the challenges they struggle to deal with as part of their everyday experience of ageing in place. Specifically, the informants highlighted contradicting information, long waiting times, and a lack of essential information as the most troublesome challenges.

#### Contradictory information

For instance, Christian, described how it was a very challenging situation for him when he was diagnosed with a blood clot in his foot and received contradicting information at the hospital about how he should care for himself after he got back home. First, a nurse told him that he should rest as much as possible and avoid physical strain. Thereafter he was told by the doctor that he should go to the pharmacy to pick up a prescribed shot and inject himself.

*Quote 14: when I had a blood clot in my foot the staff at the hospital gave me contradicting advice which was hard to deal with. The first person who examined me told me that “you need to sit very still now*,* don’t move at all or this could end very badly”*,* but then when I saw the doctor*,* he told me that I could just go to the pharmacy to get medicine and go home. That [I wasn’t sure who to trust or what was safe] really scared me.*

#### Long waiting times and a lack of relevant information

Meanwhile, Kåre highlighted how long waiting times in interactions can be very challenging in the context of ageing in place. Kåre is dependent on his inhaler which he uses every morning and every night. However, when he first started using it his vision suddenly became impaired. Everything turned grey and he could barely see anything. He could still read a little but there was no way for him to continue driving his car. He remembers thinking at the time that he thought it was weird that he would develop cataracts overnight, so he booked an appointment with his doctor who referred him to a specialist. After waiting for 1.5 years, he saw the specialist who only needed 20 min to tell him that the cortisone in his inhaler had given him cataracts in both eyes and that the problem could be solved by switching to another type of medication.

*Quote 15: Suddenly everything went grey*,* so I thought to myself “oh dear*,* am I losing my sight? What am I going to do now?“. I didn’t dare to drive so I stopped doing that. I could still read*,* at least a little bit. My doctor told me that I had developed grey cataracts*,* but I also felt like " really? in both eyes*,* almost overnight?!” I asked to see a specialist and after 1.5 years I finally got an appointment. The specialist told me that the cortisone in one of my medicines caused the cataracts and fixed it in 20 min using some ultrasound treatment*,* but still. I had to wait one and a half years.*

### Dark thoughts and depression

Another important theme in the informants’ descriptions about other health challenges concerns their mental health.

#### Struggling to keep dark thoughts at bay during lonely evenings

For instance, Solbritt described how she often has “dark thoughts”, thinks a lot about death and refrains from talking about these challenges with her close ones even if she thinks they wouldn’t mind. She also describes how these thoughts are especially hard to keep at bay during evenings and during the dark season. During the evenings she is often lonely since her husband, whom she is an informal caregiver to, goes to bed very early due to his health condition, especially during the winter months when it’s cold and very dark with only a few hours of natural light every day.

Another informant described how she gets depressed due to the lack of variation in her life where every day is the same, since she lost access to her hobby room upstairs, due to her mobility issues.

She describes how she has been deeply engaged in crafts all her life and how her crafts always have been a main source of joy in life for her. However, because the authorities only subsidize staircase elevators to essential areas such as bathrooms or bedrooms, she would have to pay for it herself which she cannot afford.

*Quote 16: since I spend so much time alone at home*,* and I don’t have the physical capacity to do much else… so*,* in a way that hobby room is my entire world*,* it’s my entire life.*

### Medications that create challenges

Another theme in the informants’ descriptions about everyday challenges of ageing in place that are related to other health problems, concerns medications that delimit their options and serve challenges while simultaneously being something that they need to manage their health. Specifically, how the participants struggle to keep track of everything they need to be mindful of in relation to their medications. This was described as especially difficult for medicines that are new to them, or medications that they only take for a short while.

#### Struggling to keep track of what to be mindful of in relation to medications

For instance, Mikkel described the challenges of dealing with his anticoagulant medications. This is challenging for him because it requires him to be very careful about what other types of medicines he takes as well as what kinds of food he ingests.

*Quote 17: and then*,* my anticoagulant medicine*,* it’s challenging because if it’s combined with the wrong food then the blood can become either too thick or too thin and if I need penicillin it can react with that too so that the blood becomes too thin. So*,* I need to be on top of that all the time.*

### Dealing with health-related fear, including fear caused by new and unknown health problems

Another main category in the informants’ descriptions about their experiences of everyday challenges in the context of ageing in place concerns general health related fear. This category of descriptions ranged from descriptions about how health related fears delimit their possibility of participating in society and social life and highlight how managing experiences of health-related fear is a considerable problem for them.

For instance, Frans described how his life is delimited because he constantly worries about his health. While he has grandchildren in both Trondheim and Oslo, he refrains from visiting them because it would entail driving for several hours which poses a risk for him if he becomes ill and needs medical assistance along the way. His fear also causes him to refrain from long trips in general which severely delimits his possibilities of participating in society and enjoying later life. In talking about this he exemplified this delimitation by describing how his fear of becoming ill has led to him having panic attacks at the airport right before he and his wife were supposed to board a plane to go on vacation.

*Quote 18: So sometimes we’ve had to turn around at the airport and go home again because of these silly fears of mine.*

Similarly, Anders describes how fear of becoming more ill also governs the way he engages in physical activity. Specifically, he describes that it is important for him to exercise with caution because he so easily catches pneumonia if he is too active. He also described that when he exercises, he tends to overextend himself and end up with an infection or inflammation. He has experienced several episodes where he has caught pneumonia and that it has later developed into sepsis which in turn has resulted in long hospital stays and he is very scared that it will happen again.

Other informants described how the discovery of new unknown symptoms happens often and that not knowing what is going on in one’s body creates a lot of fear. For instance, Mariann described that she had recently begun to develop new symptoms that she found hard to understand. Specifically, she described that she had recently begun to experience strong twitches in her hands and can’t understand why, which she finds very unsettling.

### Dealing with loneliness because of covid-related fear

Loneliness caused by covid-related fear was another important theme. This theme covered both loneliness caused by fear of covid that resulted in self-isolation, but also loneliness because of care and concern, such as when relatives refrained from visits because they were scared that it would increase the risk that their older relative would catch covid.

For instance, Ellen described that her fear of catching covid prohibited her from engaging in physical exercise because she felt it was not safe for her to participate in gym classes which she usually does twice a week. She also avoids going to the doctor’s office unless she absolutely had to and has therefore valued to opportunity to engage with her doctor digitally.

*Quote 19: I used to go a lot to the doctor’s office; it was almost like therapy but after covid I refused due to the risk of infection […] same thing with the gym. I stopped going.*

Other informants were less scared of covid and chose to not self-isolate but have nevertheless had to deal with loneliness because of the care and concern of their relatives. For instance, Kari described that she has hardly been around people for the past 2 years. Since she has so many different illnesses her children have been very scared that she will catch covid, so she has not been able to socialize with her children, grandchildren, and great grandchildren like she normally does. This has been a substantial change for her since she has always been at the centre of a very large and close-knit family.

Similarly, Christian describes how covid was challenging for him because his wife was so worried that he would catch it that she didn’t want them to socialize with other people and isolated herself to ensure his safety. They didn’t even go grocery shopping and instead chose to have their groceries delivered. They also refrained from physical contact with their family and opted to use digital solutions to stay in touch and maintain their relationships.

### The everyday challenges of ageing in a rural place

In talking about the problems and issues that they struggle with on an everyday basis the informants’ highlighted mismatches between cumbersome assistive devices and the size of their homes, a lack of alternatives when activities shut down, the manner in which the municipality organize services, keeping warm, everyday maintenance of the home, leaving the home as well as a wide variety of challenges related to winter.

### Mismatches between cumbersome assistive devices and old rural buildings

Most Norwegian people own their own home. Moreover, many of the homes in small rural local communities are very old, and some are over 100 years old. As such they do not comply with contemporary standards for buildings and are often ill suited for accommodating the often-cumbersome assistive devices that older people find themselves dependent on when they are faced with the reality of ageing in place.

### Unable to walk without support and prone to falling

Reliance on walkers and other types of assistant devices due to mobility difficulties and a history of falling was common among the informants. In talking about issues related to mobility many of the informants described that they experience a lot of pain, that they are unable to move about as they would like to and that they rely on a wide variety of medications to manage these conditions.

For instance, Britt highlighted how she and her husband would really need a ramp in front of the house due to their difficulties in traversing stairs. However, their small front yard makes it impossible to fit a ramp and still be able to park the car in front of the house to minimize the distance they must walk on ice during the winter.

*Quote 20: the staircase in front of the house*,* I could have a [ramp] there but with the steep angle of the stairs a ramp would have to stretch so far out from the house [that we wouldn’t be able to use the driveway in the front yard].*

Similarly, Gunn explained that she needs a chair with wheels on it to be able to maintain her autonomy with regards to cooking and doing other chores in the kitchen, but that the chairs available are too large and cumbersome to fit in her very small kitchen.

*Quote 21: I’d like to have one of those chairs with wheels on them*,* but it must be small because the kitchen is so small.*

Other examples included public environments. As Ellen explained.

*Quote 22: I’ve got my electric wheelchair so I can get around*,* but it’s too big so I can’t use it inside stores so I always must have someone with me and bring the other [smaller] wheelchair so they can push me even if I just need a notebook or something like that. So*,* If I am to have the freedom of being able to go out alone*,* then I can’t get into the stores.*

### Unsuitable housing, carrying water and chopping wood

In a similar vein, mismatches between the material constitution of very old homes in rural areas of Norway and the home care services that the municipalities offer can be constitutive of challenges and even barriers towards ageing in place for Norwegian older adults. For instance, one of our informants had to move to a long-term care facility because his original home was an old farm that lacked indoor plumbing and heating. When he became too frail to chop his own wood and carry water from the well to the inside, he requested help from home care services. However, because this is not considered part of care they were unable to help, and, in the end, he was awarded a place in the local long term care home to resolve the issue, despite not needing that level of care.

*Quote 23: There was not water inside so I had to get that and carry it inside*,* and I needed to chop wood*,* but I couldn’t do it anymore and it’s not something that [home care services] do so. That’s how I ended up living here instead.*

While his case is obviously not representative for most Norwegian older people it’s not a unique situation, especially in the most rural regions.

### The everyday challenges of ageing in a cold place

Winter-specific challenges were another important theme in the informants’ stories about the challenges that they struggle with. These stories highlight how winter poses challenges to ageing in place in rural Norway and creates significant barriers for older peoples’ possibilities of participating in society and ageing well independently. In talking about these challenges, both snow, ice, and the cold itself were highlighted as troublesome for the informants.

#### Removing snow and ice

In talking about their snow and ice related challenges during the winter season, most of the informants described how leaving the house becomes a challenge from October to April due to substantial amounts of snow that must somehow be dealt with. During this time of the year, their ability to leave the home hinges on their possibilities to shovel snow in front of the door, around the house and in the driveway, but also to clear snow and ice from their cars and to spread sand or gravel to decrease the risk of slipping and fallings. Sometimes clearing snow and ice is several times daily, which makes it a challenging task to solve even for those who have relatives to ask for help.

For instance, Sigrid explained that even though her son is willing to help lives very close by, dealing with the snow can still be challenging.

*Quote 24: My son lives right over there but he has a job too you know and well [he can’t be here to help us all the time] … but we do get a lot of help from him. So*,* he comes and drives the snow blower*,* and I shovel what I can.*

#### Electric wheelchairs can’t deal with the steep hills, covered with ice

Another significant challenge described by the informants is the combination of the hilly terrain surrounding them and snow or icy condition. Against this background, the informants described how they struggle during winter due to their mobility issues which make them dependent on electrical wheelchairs. As Britt described:

*Quote 25: I have my electric wheelchair but*,* it just doesn’t work with the snow.*

In this part of Norway, it is common to live on top of a hill or on a mountainside, meaning that you need to traverse steep declines and inclines to be able to participate in society by visiting friends, family, or a store. Yet, even with studded tires, electric wheelchairs are unable to manage the steep terrain in rural Norway during snowy or icy conditions, which makes it hard for older people with mobility issues to participate in society.

Due to these winter related barriers, many of the informants described how their abilities to participate in society, as well as their possibilities of enjoying an active lifestyle, and even going outside, decrease significantly between October and April.

Together, these experiences highlight how taken-for-granted activities such as going for a walk or to the store become cumbersome and sometimes even impossible for long periods of time as a heavy snowfall may make it challenging to even go out in one’s own yard on a regular basis.

## Discussion

The aim of this study was to describe the experienced needs of older people ageing in place, who have been diagnosed with Chronic Obstructive Pulmonary Disease (COPD). We have presented the key findings from an exploratory qualitative baseline interview study concerning the challenges of ageing in place with COPD in Norway. This knowledge is an important basis for future gerontechnology development, as a fit between the technology and the users’ experienced needs is crucial for uptake. In the following, we will discuss these findings and their theoretical and practical implications. We will first summarize how the identified challenges shed light on the participants’ experienced needs. Thereafter we will discuss the implications of these findings for future studies that seek to involve older people in the development of technology for ageing in place. Finally, we will address the study limitations and make suggestions for future research.

### How the identified challenges shed light on the participants experienced needs

#### COPD-related needs

Overall, the findings suggest that some needs that have previously been assumed to be central in the context of ageing in place, are not necessarily experienced as problematic by the participants in this study. One example of this is the assumption that older people living at home need support for medical adherence [28]. Participants described that they have embedded remembering and managing their medication as part of their everyday routines. This was perceived as sufficient and under their control. This finding is in line with previous research showing how medication routines are fitted into different habits [29]. Importantly then, the construction of these routines is described as solutions to the problem of remembering to take one’s medicines that already function well and there is no indication that the participants are experiencing a need or desire for other solutions that could supplement these routines or take their place.

Our findings also highlight how participants differ in how they relate to health monitoring in regard of COPD values. Those participants who had more recently gotten their diagnosis expressed a desire to monitor their health, while other actively avoided such monitoring, arguing that this kind of monitoring induced anxiety and distress. These descriptions are illustrative of how some older people with COPD experience a need for technology that allows them to register their own health values, whereas others experience a strong need to avoid knowing their own health values, and that such needs are likely to change over time. That these needs vary speaks to the need for solutions that are meant to enable the older person with COPD to take their own measurements to offer personalization, enabling users to engage with health data on their own terms. Such solutions should not take for granted that all users are interested in, or benefit from, knowing their own health values.

This finding reflects the broader heterogeneity among older adults that is often neglected, where differences in experience and preference shape how technologies are perceived and used [30].

Based on our findings, we also see that challenges related to COPD are related to broader emotional experiences. For example, participants described difficulties related to fear, and night-time anxiety due to fear caused by exacerbated symptoms This has also been found to be the case in previous studies focusing on sleep and COPD [31, 32]. Such experiences indicate a need for solutions that go beyond physiological management and addresses the psychological dimensions and troubles that are related to living with COPD.

Additionally, technologies designed specifically for COPD management were found to introduce new challenges. This problem was not due to a faulty device. The instruction manuals to portable oxygen concentrators clearly state that the device is not operational in temperatures under + 5 °C. In this way, the lack of fit between the Norwegian climate and the capacity of portable oxygen concentrators severely delimits the possibilities for older people who are ageing in place with COPD to participate in society and managing on their own. While long flexible tubes were mentioned as a tripping hazard, solutions that deal with this problem already exist. It should however be noted we did not see such solutions installed in any of the homes where there was a stationary oxygen concentrator. Falling is a huge problem so this lack of installed solutions that may prevent older people with COPD, or their family members, to trip over the long tubes, speaks to the need for solutions that may provide them with information that such solutions exist.

#### Needs not directly related to COPD

Beyond COPD-specific challenges, participants emphasized a range of everyday challenges that were not directly linked to their COPD-diagnosis but still shaped their challenges and needs in relation to ageing in place. These needs and challenges were related to other health issues they had, as well as social, organizational, and environmental factors. These include managing small but essential tasks, such as changing lightbulbs or device batteries. While these may seem like menial tasks, they are in fact incredibly important in the context of older people ageing in place. Broken lightbulbs that are not changed increase the risk of falls, and a hearing aid that does not work can both isolate and act as a potential risk for the older person. Preventing falls among older adults ageing in place is important to avoid injury, and therefore there is a need for solutions or services that support these small tasks in everyday life.

The participants also highlighted challenges related to mental and emotional strain, including loneliness, depression and persistent health-related fears. These experiences were often compounded by broader life situations, such as being informal caregivers for their frail spouses. This has been shown to be associated with an increase in depressive symptoms [33]. In addition, participants described difficulties in managing medications, particularly when dealing with new prescriptions or short-term treatments.

Health-related fear emerged as a central concern. Participants described how uncertainty about developing new health problems or complications related to COPD shaped their everyday lives and limited their engagement in physical and social activities. In this context, the need for reassurance was more prominent than the need for detailed self-monitoring. Several participants described turning to their general practitioner not primarily to obtain objective health data, but to receive authoritative and calming interpretations of their condition.

Feelings of loneliness further intensified these challenges. While covid restrictions have eased in the general population, participants described how continued vulnerability and fear of infection still led to reduced social interaction, either through self-isolation or through protective measures taken by family members. This contributed to a sustained sense of isolation and highlights how social needs remain unmet even as broader societal conditions change. Taken together, these findings point to a need for solutions that address not only physical and medical aspects of ageing in place, but also the emotional, cognitive, and social dimensions. This includes support for managing medication-related information, coping with uncertainty and anxiety, and maintaining meaningful social connections with both personal networks and healthcare providers.

Finally, many of the challenges reported by the participants were associated with the physical environment. Multimorbidity is common in older people and in addition to their COPD condition, the participants frequently described being dependent on assistive devices such as walkers, ramps, different types of wheelchairs, and staircase elevators due to mobility issues and risk of falling. This dependency is experienced as challenging to them because their assistive devices were too large for the old buildings in the rural communities where they live. Norway as well as many other European countries have a proud tradition of maintaining rather than replacing very old residential and commercial buildings as part of safekeeping the architectural and cultural legacy. In such spaces, big and cumbersome assistive devices create challenges and hindrances. As populations are increasingly ageing in place this speaks to the need for smaller, more flexible assistive devices that can fit in the smaller spaces typical for very old buildings.

A problem that is specific for the Norwegian context and other similar countries, are challenges removing snow and ice. Like mowing the lawn, removing snow is heavy and exhausting work for people with COPD. Unlike mowing the lawn, the removal of snow and ice may have to be done several times in a day which makes it hard to get help doing it, even for those who have children or other relatives who are willing to help and live nearby. In addition to increasing the risk of falls with resulting injuries and increased dependency on care services, snow and ice are also constitutive of barriers to older people’s participation in society. This speaks to the need for solutions that enable older people with COPD who live in cold climate with frequent snowfalls to deal with the challenges of removing snow. Another significant challenge described by the informants is the combination of the hilly terrain surrounding them, a dependency on electrical wheelchairs, and snowy or icy conditions. While electrical wheelchairs have studded tires, this is not enough to deal with this type of terrain. The inability for mobility devices such as electrical wheelchairs to deal with steep terrain during the long winter months makes it difficult for older people in this region to move around independently, participate in society and additionally, creates an unnecessary dependence on subsidized taxi services. This speaks to the need for assistive devices that are better equipped to deal with the road conditions during the winter season.

These findings highlight how environmental factors can significantly constrain the effectiveness of both assistive technologies and care arrangements, and point to the need for solutions that are adapted to specific geographical and climatic contexts.

#### Implications of the findings

Taken together, these findings illustrate how COPD is not necessarily the prime driver for the challenges older people with COPD face as they age in place. The participants reported four categories of challenges that they dealt with as part of ageing in place. One of these categories concern challenges related to their COPD diagnosis. However, the three other categories, meaning most reported challenges that they consider important and struggle to solve, are general problems related to the everyday experience of ageing in place. Moreover, the more general problems not directly caused by COPD were described as ongoing struggles. By contrast, the challenges they reported that were related to COPD were typically described as challenges that they already managed well. Notably, fear is a challenge that cuts across all categories. But even fear is described as manageable in descriptions that are directly related to COPD health values. From the perspective of these participants then, their need for innovative solutions does not so much concern their medical management of COPD but is more related to other struggles more related to the management of everyday life. In the context of technology development supporting ageing in place, a diagnosis-driven recruitment can lead to focusing on the wrong problems. Our study contributes to the body of literature highlighting how a focus on medical diagnosis can downplay social and emotional dimensions of everyday life. This aligns with prior research emphasizing that older adult should not be reduced to their impairments, and that too much focus on functional limitations may overshadow the broader needs that shape the use of technology [30].

In this context it needs to be recognized that the prospect of ageing in place presents different interest points as well as challenges to distinct types of stakeholders. For nation states that are seeking solutions to looming financial and staffing problems related to increased demand for care services the incentive lies in the future possibility of delegating some of the work currently performed by care professionals, to care recipients via technology. Meanwhile, older people who are already ageing in place with a given medical condition have other interests. From their perspective, the task of shouldering the responsibility of using technology to care more for themselves is neither tempting nor experienced as a pressing need. As a result, they tend to focus on other issues and challenges in talking about what they struggle with. While this does not mean that people with the same diagnosis do not share similar challenges, it does mean that it is not necessarily challenges related to their diagnoses or health that older people experience and talk about when they describe their most challenging struggles when they age in place.

This means that even projects that focus on developing technologies that are meant to cater specifically to the needs of a particular patient group are likely to benefit from pairing their focus on problems related to the medical condition in questions, with an equally strong focus on the context of everyday life.

### Limitations

This study has several limitations that are worth noting. First, participants were recruited on the basis of having a COPD diagnosis. This reflects a common practice in studies aiming to involve users in technology development but may have influenced how people framed their experiences in the interviews. However, many participants highlighted the broader everyday challenges. Secondly, the study is based on a small sample of older adults in a specific rural region and context. The findings are therefore embedded in a particular socio-material context characterized by long distances, harsh winters and features of the Norwegian system which limits transferability to other settings. Finally, the study is based on semistructured qualitative baseline interviews and captures participants’ experiences at a single point in time. As several findings suggest that needs and coping strategies may change over time, future research could benefit from a longitudinal perspective.

However, these results nevertheless offer a valuable contribution to the emerging methodological literature on the involvement of older people in the development of technologies for ageing in place.

In this context, the results offer an important perspective on the usefulness of the methodological assumption that recruiting older people with the same medical condition as co-designers will “fix” the problem of older people rejection of gerontechnology because it will lead to the discovery of homogenous needs.

As such, the results point to the importance of questioning the assumption that medical diagnoses are the most important drivers of older people needs and desires in the context of their everyday reality of ageing in place.

## Conclusions

The analysis contributes to the methodological literature on recruiting older people as co-designers of technology by challenging the assumption that medical conditions are prime drivers of older people’s needs and illustrating how this assumption may point technology developers in the wrong direction unless it is supplemented by a strong focus on the everyday experience of ageing. By challenging the assumption that medical conditions are prime drivers of older people’s needs, and how this assumption may point technology developers in the wrong direction unless it is supplemented by a strong focus on the everyday experience of ageing.

## Data Availability

The datasets generated and analysed as part of the current study are not publicly available because the format of the data does not allow for complete anonymisation.
